# Context matters: E3 ligase–ligand pairing strategies for optimized PROTAC performance

**DOI:** 10.1093/procel/pwaf107

**Published:** 2025-12-10

**Authors:** Luyao Yin, Pengcheng Shu, Xiaozhong Peng

**Affiliations:** State Key Laboratory of Common Mechanism Research for Major Diseases, Department of Biochemistry and Molecular Biology, Medical Primate Research Center, Neuroscience Center, Institute of Basic Medical Sciences, Chinese Academy of Medical Sciences, School of Basic Medicine, Peking Union Medical College, Beijing 100005, China; State Key Laboratory of Common Mechanism Research for Major Diseases, Department of Biochemistry and Molecular Biology, Medical Primate Research Center, Neuroscience Center, Institute of Basic Medical Sciences, Chinese Academy of Medical Sciences, School of Basic Medicine, Peking Union Medical College, Beijing 100005, China; State Key Laboratory of Common Mechanism Research for Major Diseases, Department of Biochemistry and Molecular Biology, Medical Primate Research Center, Neuroscience Center, Institute of Basic Medical Sciences, Chinese Academy of Medical Sciences, School of Basic Medicine, Peking Union Medical College, Beijing 100005, China; State Key Laboratory of Respiratory Health and Multimorbidity, Beijing 100005, China; Institute of Laboratory Animal Science, Chinese Academy of Medical Sciences and Peking Union Medical College, Beijing 100021, China

**Keywords:** PROTAC, E3 ligase, E3 ligand, CRBN, VHL

## Abstract

PROTACs (proteolysis-targeting chimeras) offer a revolutionary strategy to degrade proteins previously considered “undruggable.” While the importance of the target protein ligand and linker is well-established, the strategic selection of an E3 ubiquitin ligase and its corresponding ligand is an equally critical but underexplored determinant of PROTAC efficacy and selectivity. This perspective systematically analyzes how E3 ligase–ligand pairing dictates degradation outcomes across diverse biological contexts. Our analysis, incorporating head-to-head comparisons, demonstrates that no single E3 ligand is universally superior. Instead, degradation efficiency is profoundly modulated by ternary complex cooperativity, cell-type specificity, and tissue distribution. CRBN-based degraders frequently excel in hematologic malignancies, while VHL-based PROTACs show advantages in certain solid tumors. We further highlight emerging E3 ligands (e.g., from IAP, DCAF families) as promising tools to overcome resistance and expand the degradable proteome. The perspective also explores innovative frontiers, including the potential for targeting non-protein substrates and the application of PROTACs as versatile chemical knockdown tools in research. Ultimately, this paper underscores the central paradigm that “context dictates strategy” in E3 ligase selection, providing a critical framework for optimizing PROTAC design and broadening their therapeutic and research applications.

## The PROTAC revolution

Protein degradation is an evolutionarily conserved cellular process essential for the maintenance of proteostasis and the regulation of cellular homeostasis. Mammalian cells employ two major degradation systems: the ubiquitin–proteasome system (UPS) and lysosomal autophagy pathways (Nandi et al., 2006). The UPS mediates the majority of protein turnover through coordinated substrate ubiquitination and subsequent proteasomal proteolysis (Glickman and Ciechanover, 2002). This post-translational modification involves three sequential enzymatic processes: (i) ubiquitin activation catalyzed by the ubiquitin-activating enzyme (E1), (ii) ubiquitin conjugation facilitated by the ubiquitin-transferring enzyme (E2), and (iii) substrate-specific ubiquitin ligation executed by the ubiquitin ligase (E3). Notably, the human genome encodes over 600 distinct E3 ubiquitin ligases, which confer substrate specificity through their unique substrate recognition domains (Clague et al., 2015). This enzymatic cascade ensures precise spatiotemporal control of protein degradation, maintaining cellular equilibrium through selective elimination of misfolded or regulatory proteins.

**Figure 1.**
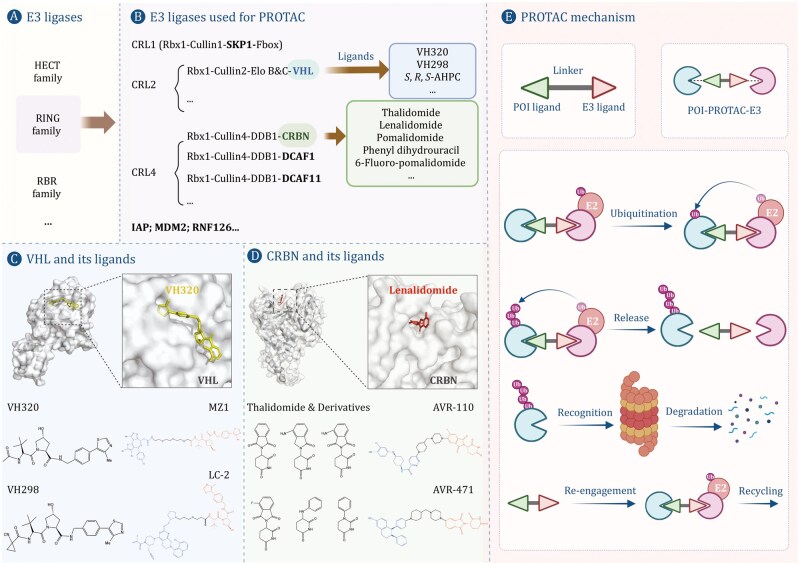
**E3 ligase toolbox and mechanism of PROTAC-mediated degradation**. The schematic outlines key E3 ubiquitin ligase complexes (e.g., CRL2VHL, CRL4CRBN) and their small-molecule ligands (e.g., VH032 for VHL, Pomalidomide for CRBN) used in PROTAC design. A PROTAC molecule bridges a protein of interest (POI) and an E3 ligase via a linker, inducing ternary complex formation. This leads to polyubiquitination and proteasomal degradation of the POI, resulting in functional consequences such as apoptosis.

Proteolysis-targeting chimeras (PROTACs) represent a groundbreaking therapeutic strategy in targeted protein degradation that has gained significant traction over the past decade. Before 2015, fewer than 100 publications on PROTACs (including related terms such as “proteolysis-targeting chimeras” or “PROTAC degraders”) were indexed in databases such as PubMed, a number that has since surged to over 3,400 entries by October 2025. Structurally, PROTACs consist of three distinct functional components: a ligand that binds the protein of interest (POI), a ligand that recruits an E3 ubiquitin ligase, and a connecting linker moiety. Upon entry of the PROTAC molecule into the cell, the target protein ligand specifically binds to POI, and the E3 ligase ligand recruits the E3 ubiquitin ligase, forming a POI-PROTAC-E3 ligase ternary complex. The ternary complex ubiquitinates the target protein, and the ubiquitinated target protein is subsequently degraded by the proteasome (Fig. 1).

## The molecular blueprint of PROTACs and the evolution of E3 ligands

The PROTAC concept was originally proposed and validated in 2001 by Prof. Crews (Yale University) and Prof. Deshaies at Caltech (Sakamoto et al., 2001). They successfully developed the first peptide PROTAC/bioPROTAC, with the phosphopeptide segment Ile-Pro-Pro (IPP) as a well-defined ligand for the E3 ubiquitin ligase Skp1–Cullin1–F-box complex, and the ovalbumin (OVA) covalently bound to methionine aminopeptidase 2 (MetAP2). The OVA-IPP PROTAC achieves ubiquitination and degradation of MetAP2. Despite favorable biocompatibility, low cytotoxicity, and efficient target degradation, the utility of peptide PROTAC has been hampered by its high molecular weight, poor cell membrane permeability, and chemical instability. In 2008, Schneekloth et al. developed the first small-molecule PROTAC by exploiting the high-affinity interaction between the E3 ligase MDM2 and the imidazoline derivative Nutlin (Schneekloth et al., 2008). By conjugating Nutlin to a defined androgen receptor (AR) ligand SARM, they generated a chimeric molecule capable of inducing AR degradation, thereby establishing the feasibility of cell-permeable small-molecule PROTACs.

Molecular glue degraders (MGDs), considered a distinct yet complementary therapeutic modality of PROTACs, are typically small molecules that stabilize protein–protein interactions between an E3 ligase and its substrate (Hanzl and Winter, 2020). Thalidomide, one of the most characterized MGDs, exhibits multi-target activity by directly engaging several proteins. The identification of CRBN (cereblon) as a direct target of thalidomide in 2010 significantly advanced the MGD paradigm (Ito et al., 2010). CRBN serves as the substrate recognition component of the Cullin 4 RING ligase (CRL4) complex. Thalidomide binding to CRBN promotes recruitment of non-physiological substrates, leading to their proteasomal degradation. Thalidomide and its analogs, known immunomodulatory imide drugs (IMiDs), are well-established ligands for CRBN-based PROTACs (Chamberlain et al., 2019). The targets of PROTAC include overexpressed oncoproteins (e.g., ER, AR, BRD), disease-associated fusion proteins (e.g., BCR–ABL), mutant oncoproteins (e.g., KRAS), and various kinases (e.g., CDKs, RTKs) implicated in cancer, cardiovascular disorders, immune dysregulation, neurodegenerative conditions, and viral infections (Gough et al., 2024; Ji et al., 2025a; Lai et al., 2016; Raina et al., 2016; Shi et al., 2022; Snyder et al., 2025; Wang et al., 2024). Although only a limited number of PROTACs have advanced into clinical trials, numerous compounds targeting diverse POIs have been patented. Among these, ARV-471, an ER-targeting PROTAC for the treatment of ER^+^/HER2^−^ breast cancer, has progressed to Phase III clinical trials (Gough et al., 2024).

The selection of E3 ligases and their corresponding ligands is crucial for the efficient formation and function of the POI-PROTAC-E3 ternary complex. Even with similar linkers and POI ligands, the choice of E3 ligase ligand can lead to significant differences in degradation efficiency and selectivity. This perspective systematically examines the impact of E3 ligase and ligand selection on the efficacy of PROTAC molecules, discusses current challenges in PROTAC technology, and outlines future directions for its application.

## The canonical E3 ligase toolkit: CRBN and VHL

Certain single-subunit E3 ligases harbor intrinsic substrate-binding domains within their polypeptide sequences. These enzymes, such as MDM2, which facilitates p53 degradation, can directly engage substrates and catalyze their ubiquitination without requiring additional protein partners (Haupt et al., 1997). In contrast, multi-subunit ubiquitin ligase complexes often employ dedicated substrate recognition subunits. A prominent example is the Cullin-RING ligase (CRL) family, the largest group of E3 ubiquitin ligases in humans, which plays critical roles in diverse cellular homeostasis processes, including oxidative stress response, signal transduction, DNA damage repair, and cell cycle regulation (Nguyen et al., 2017). The CRL E3 complex consists of four basic subunits: Cullin scaffold proteins (Cullin 1, 2, 3, 4A, 4B, 5, 7, or 9), RING finger domain proteins (RBX1/ROC1 or RBX2/ROC2/SAG), adaptor proteins (SKP1, ElonginB, ElonginC, or DDB1), and substrate receptor proteins (F-box, VHL [von Hippel–Lindau] box, BTB box, DCAF, SOCS box, or Fbw8) (Richardson et al., 2002). These substrate receptor proteins or their interacting proteins are typically regarded as E3 ligase recruitment elements (ie, E3 ligands) and have been engineered for incorporation into PROTAC molecules with tailored properties. For application in PROTAC design, E3 ligases must fulfill several criteria: (i) high endogenous expression levels, (ii) structural flexibility enabling ubiquitination of diverse protein substrates with varying primary sequences and tertiary folds, and (iii) the ability to generate efficient polyubiquitin chains—­particularly K11- and K48-linked linkages—that are preferentially recognized by the proteasome.

The development of E3 ligands has historically been challenging due to the absence of conventional druggable pockets in E3 ligases. The discovery of E3 ligands necessitates targeting protein–protein interactions, which exhibit large, flat interfacial surfaces, making their engagement with drug-like compounds particularly challenging (­Galdeano, 2017). Between 2010 and 2014, however, breakthrough discoveries identified high-affinity, drug-like small-molecule ligands for CRL4^CRBN^ and CRL2^VHL^, with their binding modes elucidated at atomic resolution via crystallography (Buckley et al., 2012a; Fischer et al., 2014; ­Galdeano et al., 2014). These ligands exhibit well-defined binding poses, high specificity and affinity for their cognate E3 ligases, and favorable physicochemical properties (Diehl and Ciulli, 2022; Testa et al., 2018). Advances in high-throughput screening and bioinformatic approaches have since expanded the repertoire of E3 ligands suitable for PROTAC design, including MDM2 (Hines et al., 2019), IAP (Naito et al., 2019), RNF114 (Luo et al., 2021), DCAF11 (Zhang et al., 2021), DCAF16 (Zhang et al., 2019), and KEAP1 (Wei et al., 2021). Despite these developments, CRBN and VHL ligands remain the most extensively validated and widely utilized E3 recruiters in PROTAC applications.

### CRL4^CRBN^: from thalidomide to a cornerstone of targeted degradation

CRBN functions as a substrate receptor within the CRL4 E3 ubiquitin ligase complex and belongs to the DCAF (DDB1- and CUL4-associated factor) family. The CRL4 subfamily additionally comprises approximately 70 DCAF proteins, which recruit specific substrates to the CRL4 E3 complexes (Jang et al., 2021). These DCAF proteins not only determine the specificity of protein ubiquitination but also regulate the spatiotemporal dynamics of CRL activity. Thalidomide, the most extensively characterized small-molecule ligand for CRBN, contains a phthalimide ring and a glutarimide moiety (Fig. 1). Initially marketed as a sedative-hypnotic agent, it was withdrawn in the 1960s due to severe teratogenicity (Vargesson, 2015). Nonetheless, owing to its potent biological properties, thalidomide was repurposed for the treatment of multiple myeloma (MM; Richardson et al., 2002). Subsequent derivatives, lenalidomide and pomalidomide, also demonstrate potent anticancer activity (Bartlett et al., 2004). The identification of CRBN as a direct target of thalidomide in 2010 revealed that these compounds act as “molecular glues,” redirecting CRL4^CRBN^ to neo-substrates for ubiquitination and degradation (Ito et al., 2010). This mechanistic insight solidified their utility as E3-recruiting ligands in PROTAC design, with the “POI inhibitor–linker–thalidomide/derivative” architecture emerging as a predominant strategy in CRBN-based PROTAC development.

The first CRBN-based PROTAC, dBET1, was developed by conjugating the BET inhibitor JQ1 to a thalidomide derivative. This chimera induced CRBN-dependent degradation of BET proteins in MM and acute myeloid leukemia (AML) cell lines and suppressed tumor growth in murine xenograft models (Winter et al., 2015). Since then, over 200 CRBN-based PROTACs have been designed to target an array of pathogenic proteins, including protein kinases (e.g., ALK, PI3Kδ, BCR-ABL), transcriptional regulators (e.g., BET proteins, BCL family members, STAT3), and nuclear receptors (e.g., AR) (He et al., 2020; Ji et al., 2025a; Lai et al., 2016; Raina et al., 2016; Snyder et al., 2025; Yan et al., 2021; Yuan et al., 2025). These compounds hold therapeutic potential in oncology, cardiovascular disorders, neurodegenerative diseases, and beyond (Wang et al., 2021). It is noteworthy that several CRBN-based PROTACs, including ARV-471, ARV-110, ARV-766, and NX-2127 (Fig. 1), have advanced to clinical trial phases (Gough et al., 2024; Montoya et al., 2024; Snyder et al., 2024, 2025).

### CRL2^VHL^: from tumor suppressor to potent degradation recruiter

The *VHL* gene was first discovered in 1993 (Latif et al., 1993). Mutations in this gene cause VHL syndrome, an autosomal dominant disorder characterized by heightened susceptibility to various tumors, including retinoblastoma and hemangioblastoma (Kaelin, 2003). Subsequent research established that the VHL protein serves as a substrate recognition subunit within the CRL2 ubiquitin ligase complex (Cardote et al., 2017). Its most extensively characterized substrate is hypoxia-inducible factor 1α (HIF-1α). Initially, scientists designed a PROTAC targeting AR using the seven-amino acid sequence of HIF-1α (the smallest domain recognized by VHL) as the VHL-binding agent. However, due to its large molecular weight, it exhibited poor cellular permeability (­Schneekloth et al., 2004). Since high-throughput and virtual screening methods failed to identify viable VHL-binding compounds, researchers from the Ciulli and Crews laboratories modeled the binding pattern of VHL with HIF-1α and designed the first VHL small-molecule ligand with a micromolar dissociation constant, using hydroxyproline (Hyp) as the core and adding groups to the left-hand side (LHS) and right-hand side (RHS) (Buckley et al., 2012b). This breakthrough was followed by the Ciulli group’s development of VH032 (Fig. 1), a second-generation VHL ligand with improved affinity and lipophilicity (Galdeano et al., 2014), and later VH298 (Soares et al., 2018). In recent years, extensive chemical optimization of these scaffolds has been pursued to enhance binding affinity, cellular permeability, and degradation specificity. The determination of the crystal structure of the VHL-based PROTAC MZ1 (Fig. 1) provided the first visual evidence of how PROTAC molecules mediate ternary complex formation between the POI and the VHL E3 ligase (Zengerle et al., 2015). Among the resulting therapeutic advances, the VHL-based PROTAC DT-2216 has entered clinical trials for anticancer therapy (He et al., 2020). It is noteworthy that the majority of published VHL-directed PROTACs to date have utilized VH032 as the E3-recruiting moiety.

A conventional strategy in PROTAC development involves conjugating established binders of POI to CRBN or VHL ligands via synthetic linkers. This enables the construction of diverse PROTAC libraries for structure–activity relationship (SAR) studies. Candidates are then evaluated across multiple cellular models and treatment conditions to identify leads with optimal efficacy. Many POI binders used in these constructs are clinical-stage inhibitors themselves, which may reduce the risk of off-target effects. The structural architecture of the linker—including its length, rigidity, flexibility, hydrophilicity, lipophilicity, and sites of attachment—critically influences the stability and physicochemical properties of PROTAC molecules. Linkers are broadly categorized as flexible, rigid, or functionally specialized (Troup et al., 2020). Ongoing research continues to advance the development of controllably engineered PROTACs through rational linker design.

## E3 ligase selection: a context-dependent strategic decision

The three core structural components of PROTAC molecules collectively determine their functional efficacy. The POI ligand provides the foundational binding affinity for the target, while subtle modifications in linker positioning or length frequently yield substantial enhancements in degradation efficiency—sometimes even enabling transition from inactive to active states. Strategic selection of E3 ligase ligands, however, provides critical opportunities to overcome efficacy limitations and adapt PROTAC systems for diverse biological contexts.

**Figure 2.**
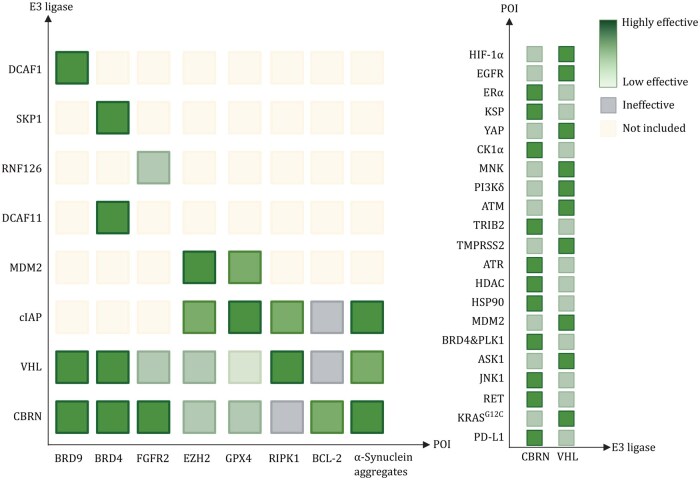
**Heatmap analysis of target degradation efficiency dictated by E3 ligase selection**. Heatmap analysis of PROTAC efficacy across E3 ligases and target proteins. Performance is highly dependent on the specific E3-POI pair. Degradation efficiency is visualized by a green color gradient (dark green: high degradation; light green: moderate degradation; pale yellow: not included; gray: no degradation). Data are derived from head-to-head comparisons across diverse cellular models (see Table 1 for details).

Our systematic review compares the degradation efficiency of PROTACs targeting identical POIs but recruiting distinct E3 ligases, as documented in existing literature. The analytical methodology for Table 1 is as follows: (i) we identified studies that directly compared PROTACs for a specific POI using different E3 ligands; (ii) from each study, we extracted the PROTAC molecule reported to be the most effective; (iii) the E3 ligase or ligand recruited by this top-performing PROTAC was designated as “Potent”; and (iv) we also summarized the POI ligands and cellular models used in these comparative evaluations. Based on the tabulated analysis, we have generated a degradation heatmap (Fig. 2) to visually represent the context-dependent efficacy of different E3 ligands.

**Figure 3.**
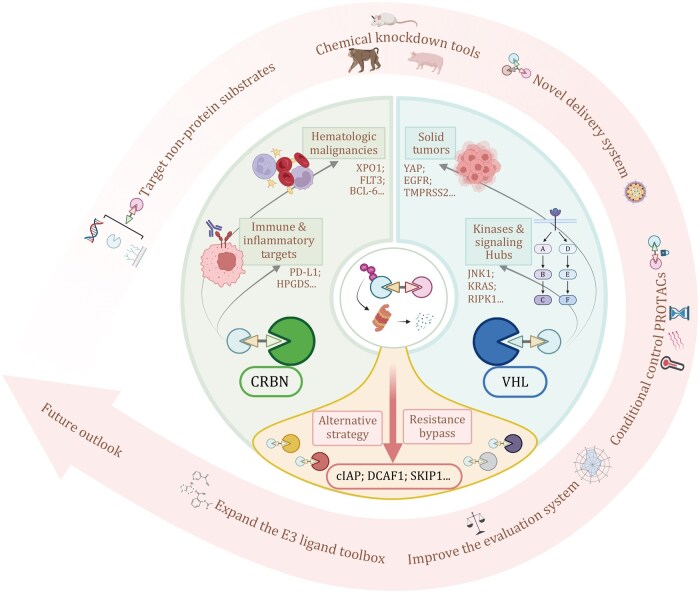
**Strategic selection of E3 ligases dictates the context-specific efficacy of PROTAC technology**. This schematic summarizes the paradigm of matching E3 ligase recruiters with specific pathological contexts and target classes to optimize PROTAC performance. The canonical E3 ligases CRBN and VHL serve as the primary hubs, demonstrating preferential efficacy in hematologic malignancies/immune targets (e.g., XPO1, FLT3, PD-L1) and solid tumors/kinase signaling hubs (e.g., EGFR, KRAS, TMPRSS2), respectively. The expansion to non-canonical E3 ligases (e.g., cIAP, DCAF1, SKP1) provides an essential alternative strategy to overcome intrinsic or acquired resistance to CRBN- or VHL-based degraders. Collectively, this evolving E3 ligase toolkit positions PROTACs as versatile “chemical knockdown tools” with a broad therapeutic and research outlook.

**Table 1.** Systematic comparison of E3 ligand efficiency in PROTAC-mediated target degradation across cellular models.

Compared E3 ligands		POI ligand	POI	Cell lines used in the experiment	Potent	References
**CRBN ligands**	Lenalidomide Pomalidomide	pan-FGFR inhibitor (AZD454)	FGFR1	MCF-7 (human breast cancer cell line)	Pomalidomide	Wang et al. (2025)
	A benzamide-based structure Pomalidomide	XPO1 inhibitor (CBS9106)	XPO1	MV4-11, MOLM-1 (acute myeloid leukemia cell lines)	The benzamide-based structure	Chen et al. (2025)
	Lenalidomide Thalidomide	SOS1 inhibitor (BI3406)	SOS1	A549 (human non-small cell lung cancer cell line) MIA-PACA2 (KRAS^G12C^), HPAF2 (KRAS^G12D^) (human pancreatic cancer cell lines)	Lenalidomide	Li et al. (2024a)
	Lenalidomide Thalidomide	BCL6 inhibitor (14j)	BCL6	SU-DHL-4, SU-DHL-6, DOHH2, OCI-Ly1 (human diffuse large B-cell lymphoma cell line) Mouse PDX model	Thalidomide	Mi et al. (2024)
	Thalidomide Lenalidomide	FLT3 inhibitor (Gilteritinib)	FLT3	MV4-11 (acute myeloid leukemia cell line)	Lenalidomide	Liu et al. (2024b)
	Phenyl glutarimide 6-Fluoro-pomalidomide	H-PGDS inhibitor (TFC-007)	H-PGDS	KU812 (human basophilic leukemia cell line)	Phenyl glutarimide	Osawa et al. (2023)
	Thalidomide Fluorophenyl-amino-glutarimide Phenyl glutarimide Phenyl dihydrouracil A bridged glutarimide 3-Dihydrouracilyl indazole derivative	KRAS^G12D^ inhibitor (MRTX1133)	KRAS^G12D^	AsPc-1, GP2D, PK-59, ASG (KRAS^G12D^ cancer cell lines)	3-dihydrouracilyl indazole derivative	Ji et al. (2025b)
**VHL ligands and CRBN ligands**	VHL ligand CRBN ligands (lenalidomide, pomalidomide)	PD-L1 inhibitor (A56)	PD-L1	HCC-827 (human non-small cell lung cancer cell line) MC38 mouse colon cancer model	Pomalidomide	Zhang et al. (2024b)
	VHL ligand (*S*,*R*,*S*-AHPC) CRBN ligands (lenalidomide, pomalidomide)	KRASG12C inhibitor (AMG-510)	KRAS^G12C^	NCI-H358 (human non-small cell lung cancer cell line)	VHL ligand	Zhang et al. (2023)
	VHL ligand CRBN ligands (lenalidomide, thalidomide)	RET inhibitor (LOXO-292)	RET	Ba/F3 (murine pro-B lymphocyte cell line) Xenograft mouse model	CRBN ligand (lenalidomide)	Hualong et al. (2025)
	VHL ligand CRBN ligand (thalidomide)	JNK1 inhibitor (P1)	JNK1	BEAS-2 (human normal pulmonary epithelial cell line)	Thalidomide	Guo et al. (2025)
	VHL ligand (VH032) CRBN ligand (thalidomide)	ASK1 inhibitor (Selonsertib fragment)	ASK1	HepG2 (human hepatoma cell line) Methionine choline-deficient diet-induced murine model	VHL ligand	Sarkar et al. (n.d.)
	CRBN ligand (pomalidomide) VHL ligand (*S*,*R*,*S*-AHPC)	Dual inhibitor of BRD4 and PLK1 (BI-2536)	BRD4 and PLK1	K562 (human chronic myeloid leukemia cell)	CRBN ligand (pomalidomide)	Song et al. (2025)
	CRBN ligand (thalidomide) VHL ligand	MDM2 binder (GAA)	MDM2	MCF-7, MDA-MB-231 (human breast cancer cell lines) Zebrafish PDX model	VHL ligand	Li et al. (2024b)
	VHL ligand CRBN ligands (lenalidomide, pomalidomide)	HSP90 inhibitor (SNX-5422 and SNX-2112)	HSP90	SiHa (human endometrial carcinoma cell line) Mouse PDX model	Lenalidomide	Liang et al. (2024)
	VHL ligand CRBN ligands (thalidomide derivative, N-aryl glutarimide, phenyl dihydrouracil derivative)	HDAC8 inhibitor	HDAC	Jurkat (human acute T-cell leukemia cell line) THP-1 (acute myeloid leukemia cell line) HCT116 (human colon cancer cell line) A549 (human non-small cell lung cancer cell line)	CRBN ligand (N-aryl glutarimide)	Zhao et al. (2024a)
	CRBN ligands (thalidomide, lenalidomide, other glutarimides) VHL ligand	ATR inhibitor (VE-821 and VE-822)	ATR	MIAPaCa-2 (human pancreatic carcinoma cell line)	Lenalidomide	Alfayomy et al. (2024)
	CRBN ligands (thalidomide, lenalidomide, pomalidomide) VHL ligand (*S*,*R*,*S*-AHPC-Me, VH032)	TMPRSS2 inhibitor (Camostat)	TMPRSS2	Calu-3 (human lung adenocarcinoma cell line) HT-29 (human colorectal adenocarcinoma cell line)	VHL ligand (*S*,*R*,*S*-AHPC-Me)	Yang et al. (2024)
	VHL ligand CRBN ligands (thalidomide, pomalidomide)	TRIB2 inhibitor	TRIB2	PC3 (human prostate cancer cell line)	CRBN ligand (pomalidomide)	Wen et al. (2025)
	CRBN ligand (lenalidomide) VHL ligand (VH032-Boc)	ATM inhibitor (Mei derivative)	ATM	SW620, SW480 (human colorectal cancer cell lines) K562 (human chronic myeloid leukemia cell)	VHL ligand	Liu et al. (2024a)
	CRBN ligand (lenalidomide) VHL ligand (VH032)	PI3Kδ inhibitor	PI3Kδ	SU-DHL-6, Pfeiffer (human diffuse large B-cell lymphoma cell line)	VHL ligand	Yuan et al. (2025)
	CRBN ligand (lenalidomide) VHL ligand	Derivative of MNK inhibitor ETC-206 (DS33059)	MNK	TMD-8 (diffuse large B-cell lymphoma cell lines) A549 (human non-small cell lung cancer cell line)	VHL ligand	Sun et al. (2024)
	CRBN ligand (pomalidomide, the benzamide type structure) VHL ligand	CK1α, CDK7 and CDK9 multiple-targeted inhibitor (A86)	CK1α	MV4-11 (human acute myeloid leukemia cell line)	CRBN ligand (the benzamide type structure)	Wang et al. (2024)
	CRBN ligand (pomalidomide) VHL ligand	YAP binder (NSC682769)	YAP	NCI-H226 (human lung squamous cell carcinoma cell line) Huh7 (human hepatocellular carcinoma cell line)	VHL ligand	Zhou et al. (2024)
	CRBN ligand (pomalidomide) VHL ligand	ATR inhibitor (AZD6738)	ATR	LoVo (human colorectal adenocarcinoma cell line) HCT116 (human colorectal carcinoma cell line)	CRBN ligand (Pomalidomide)	Huang et al. (2024)
	CRBN ligand (thalidomide) VHL ligand	KSP inhibitor	KSP	HCT-116 (human colorectal carcinoma cell line) Xenograft mouse model	CRBN ligand (thalidomide)	Zhao et al. (2025)
	CRBN ligand (thalidomide, lenalidomide) VHL ligand	ERα-selective estrogen receptor degrader (SAR439859)	ERα	MCF-7 (human breast cancer cell line)	CRBN ligand (lenalidomide)	Yang et al. (2025)
	CRBN ligand (pomalidomide) VHL ligand	EGFR inhibitor (Dacomitinib)	EGFR	HCC-827 (human non-small cell lung cancer cell line) Mouse xenograft tumor model	VHL ligand	Shi et al. (2022)
	CRBN ligand (4-Flusalidomide) VHL ligand	HIF-1α inhibitor (IDF-11774)	HIF-1α	MDA-MB-231 (human breast cancer cell lines) HepG2 (human hepatocellular carcinoma cell line)	VHL ligand	Li et al. (2024c)
**Other ligands**	DCAF1 ligand (OICR-8268) VHL ligand (VHL4) CRBN ligand (pomalidomide)	BRD9 bromodomain binder (BI-9564)	BRD9	HEK293T (human embryonic kidney cell line)	Equally comparable	Schröder et al. (2024)
	SKP1 ligand (EN884 derivative) CRBN ligand VHL ligand (VH032)	BET inhibitor (JQ1)	BRD4	293 (human embryonic kidney cell line) MDA-MB-231 (human breast cancer cell lines)	Equally comparable	Hong et al. (2024)
	VHL ligand IAP ligand CRBN ligand (pomalidomide)	Pan-MLK inhibitor (CEP1347)	MLK3	A375 (human malignant melanoma cell line) HCC1806 (human breast cancer cell lines)	VHL ligand	Karpińska et al. (2024)
	VHL ligand (VH298) CRBN ligand (pomalidomide) cIAP ligand (CST530)	Non-selective BCL-2/BCL-xL inhibitor (Venetoclax derivative) Dual MCL-1/BCL-2 inhibitor (Nap-1 derivative)	BCL-2	MM.1S (human multiple myeloma cell line) HeLa (human cervical adenocarcinoma cell line)	CRBN ligand (pomalidomide)	Bricelj et al. (2024)
	CRBN ligand (pomalidomide) VHL ligand (VH032) cIAP1 ligand (bestatin)	Aggregation inhibitor of α-synuclein (sery308)	α-Synuclein aggregates	HEK293T (human embryonic kidney cell line) SH-SY5Y (human neuroblastoma cell line) *C. elegans* strain NL5901	CRBN ligand (Pomalidomide)	Tong et al. (2023)
	CRBN ligand (pomalidomide) VHL ligand (VH032) cIAP1 ligand (bestatin)	Aggregation inhibitor of α-synuclein (sery384)	α-Synuclein aggregates	H293T (human embryonic kidney cell line)	cIAP1 ligand	Wen et al. (2023)
	CRBN ligand (thalidomide) VHL ligand (*S*,*R*,*S*-AHPC-Me) cIAP ligand (LCL161)	RIPK1 inhibitor	RIPK1	I2.1 (human acute T-cell leukemia cell line) Hepa1-6 (mouse hepatocellular carcinoma cell line)	VHL ligand	Zhang et al. (2025)
	CRBN ligand (thalidomide 4-fluoride) MDM2 ligand (nutlin-3) VHL ligand (VH032) cIAP ligand (B4, B5)	GPX4 inhibitor (RSL3)	GPX4	HT1080 (human fibrosarcoma cell line)	cIAP ligand	Song et al. (2024)
	CRBN ligand (thalidomide) VHL ligand (VH032 derivatives) cIAP ligand (beta statin derivatives) MDM2 ligand (nutlin-3)	EZH2 inhibitor (EPZ6438)	EZH2	SU-DHL-6, HBL-1 (human diffuse large B-cell lymphoma cell lines)	MDM2 ligand	Xie et al. (2023)
	CRBN ligand (thalidomide) VHL ligand RNF126 ligand	pan-FGFR inhibitor (LY2874455)	FGFR2	KATO III (human gastric carcinoma cell line) MCF-7 (human breast cancer cell lines) Huh-7 (human hepatocellular carcinoma cell line) BXPC3 (human pancreatic adenocarcinoma cell line) Ba/F3 (mouse pro-B lymphocyte cell line)	CRBN ligand (thalidomide)	Hu et al. (2024)
	DCAF11 ligand (alkenyl oxindole derivative) CRBN (pomalidomide) VHL (VH032)	BET inhibitor (JQ1)	BRD4	MCF7, HCC1937, MDA-MB-231, MDA-MB-468 (human breast cancer cell lines)	Equally comparable	Zhao et al. (2024b)

This comprehensive table summarizes head-to-head evaluations of PROTACs designed to degrade identical protein targets but recruiting distinct E3 ubiquitin ligases via varied ligand chemistries.

Our comparisons encompass both widely utilized CRBN/VHL ligands and emerging alternatives (DDB1, DCAF1, SKP1, IAP, cIAP, MDM2, and DCAF11 ligands). These investigations span multiple oncoprotein targets across hematologic and solid malignancies, employing varied cancer cell models. Most studies systematically explore linker variations and E3 ligand modifications to construct PROTAC libraries, evaluating target degradation and anti-proliferative effects across single or multiple cell lines. Notably, direct biophysical validation of ternary complex formation remains scarce, with only limited support from computational modeling (Table S1). A limited subset extends to SAR profiling, pharmacokinetic characterization, and membrane permeability assessments, with only rare examples validating *in vivo* efficacy through animal models (murine patient-derived xenografts, zebrafish, *C. elegans*).

As summarized in Table 1, the results clearly show that the selection of E3 ligands does not have a universal optimal solution, but is strongly dependent on the specific context of the target protein, the cell type, and even the disease model.

### Head-to-head comparison of CRBN versus VHL ligands

Among CRBN-based ligands (thalidomide, lenalidomide, pomalidomide, and derivatives), none exhibits a consistent advantage. For instance, lenalidomide proves more effective in directing CRBN to degrade FLT3 (Liu et al., 2024b) and SOS1 (Li et al., 2024a), whereas pomalidomide exhibits superior activity against FGFR1 (Wang et al., 2025) and TRIB2 (Wen et al., 2025). Thalidomide, in turn, emerges as the preferred recruiter for BCL6 degradation (Mi et al., 2024). A case in point is the development of a novel 3-dihydrouracilyl indazole derivative for targeting KRAS^G12D^, which underscores the critical importance of meticulous chemical optimization of the E3 ligand itself (Ji et al., 2025b). Though fluorination and heterocyclic modifications can enhance specificity—albeit with trade-offs in potency.

The broader tissue distribution and lower molecular weight of CRBN ligands have fueled greater research investment; however, VHL-based PROTACs demonstrate comparable therapeutic potential. For identical POIs, VHL-based PROTACs achieved superior degradation in 50% of cases, with comparable efficacy observed against targets like BRD4 (Hong et al., 2024; Zhao et al., 2024b). VHL ligands exhibit exceptional performance against KRAS^G12C^ (Zhang et al., 2023) and YAP (Zhou et al., 2024), while CRBN ligands (particularly pomalidomide) show an advantage against PD-L1 (Zhang et al., 2024b) and XPO1 (Chen et al., 2025). CRBN-based agents dominate in hematologic malignancies and immune checkpoint targeting, whereas VHL-based compounds excel in solid tumors (e.g., KRAS-mutated cancers and kinase targets).

### Beyond CRBN and VHL: opportunities with emerging E3 ligases

Compared to non-CRBN/VHL ligands, CRBN/VHL-based PROTACs generally demonstrate superior efficacy in the majority of cases. Nonetheless, IAP/cIAP ligands (e.g., LCL161, Bestatin) display advantages in apoptotic pathways (BCL-2, RIPK1) (Bricelj et al., 2024; Zhang et al., 2025) and neurodegenerative targets (α-synuclein) (Wen et al., 2023). Other ligands (MDM2, SKP1) show restricted but notable activity against specific targets (EZH2, BRD4) (Hong et al., 2024; Xie et al., 2023), likely reflecting limited optimization due to less extensive characterization. This landscape will undoubtedly evolve with improved structural and mechanistic understanding of these E3 systems.

The limitations of CRBN/VHL systems highlight the value of alternative E3 recruiters.

A primary concern is the emergence of resistance. Sperling et al. reported that thalidomide analogues exhibit a specific order during multi-substrate degradation, with substrates that have higher affinity and/or higher abundance can outcompete other substrates (Sperling et al., 2019). Substrates interact and compete with each other. The induced overexpression of one substrate reduces the degradation of other substrates, leading to resistance in cellular models. Song et al. demonstrated that a dual-targeting PROTAC designed to simultaneously engage BRD4 and PLK1 induced near-complete degradation of BET family proteins and PLK1 at nanomolar concentrations. However, kinetic profiling revealed a pronounced temporal disparity in degradation efficiency: BET protein degradation occurred rapidly within 4 h, whereas PLK1 proteolysis requiring 36 h (Song et al., 2025).

The widespread adoption of CRBN-based PROTACs is partly attributable to the extensive clinical validation of IMiDs, yet this also elevates the risk of resistance ­emergence. Clinical cohort studies have identified CRBN ­mutations in up to one-third of patients with pomalidomide-resistant diseases, including missense mutations within the thalidomide-analogue binding domain (Kortüm et al., 2016). Beyond directly impairing protein degradation, cancer cells may also acquire mutations in downstream signaling pathways to circumvent the effects of targeted protein degradation. For instance, in some patients with myelodysplastic syndrome (MDS), lenalidomide induces p53-mediated cell death by degrading CK1α. However, MDS patients undergoing lenalidomide treatment exhibit an increased mutation frequency in TP53—the gene encoding p53—leading to acquired resistance to lenalidomide therapy (Järås et al., 2014; Krönke et al., 2015; Lodé et al., 2018).

The absence of VHL in renal carcinoma cells confers inherent resistance to VHL-based PROTACs, while diminished clinical response due to CRBN-mediated resistance has been documented in patients receiving IMiDs therapy. In this context, DCAF1-based PROTACs represent a viable alternative to CRBN-based PROTACs. The DCAF1-BRD9 PROTAC (designated DBr-1) effectively degrades BRD9 in cell lines lacking VHL expression (Schröder et al., 2024). Furthermore, certain pathogenic proteins exhibit low susceptibility to degradation via CRBN- or VHL-mediated mechanisms. In a PROTAC design targeting GPX4 degradation, compounds recruiting cIAP or MDM2 achieved significantly higher degradation efficacy (80% and 60%, respectively) compared to those utilizing CRBN or VHL ligands (40%–50% and 30%–40%, respectively) (Song et al., 2024).

### Cell, tissue, and species specificity: intrinsic determinants of efficacy

Moreover, the intrinsic properties of the cell lines employed for evaluation and the structural and functional characteristics of the POI itself profoundly influence the selectivity and efficacy of PROTAC molecules. Meyers et al. utilized the DDB1-directed ligand MM-02-57 as an E3 recruiter and the BET inhibitor JQ1 as a POI-targeting moiety to synthesize a series of PROTACs directed against BET proteins. Notably, in HEK293T cells, all six resultant PROTAC constructs selectively degraded the short isoform of BRD4, while sparing the long isoform—a finding inconsistent with prior studies employing VHL- or CRBN-based ligands to degrade BRD4. However, both isoforms were effectively degraded in MDA-MB-231 breast cancer cells, suggesting that the observed isoform selectivity may reflect cell line-specific biological contexts rather than an inherent incapacity of DDB1-based PROTACs to engage the long BRD4 isoform within a ternary complex (Meyers et al., 2024). A separate study targeting BCL-2 reported potent degradation in HeLa cells, yet demonstrated no appreciable activity in a range of hematologic malignancy cell lines (Wang et al., 2019).

These observations underscore that the expression levels and compositional dynamics of the entire UPS—including the substrate, substrate receptors, and E3 complex components—collectively modulate PROTAC-induced degradation efficiency. Such variability becomes even more pronounced when considering tissue-specific selectivity. Zorba et al. demonstrated that a CRBN-based PROTAC (designated PROTAC 10) mediated strikingly divergent BTK degradation efficacy in rat spleen versus lung tissues, despite comparable compound distribution and biodistribution profiles (Zorba et al., 2018). Additionally, species-specific differences influence PROTAC optimization. For instance, the linker length optimal for degrading human RIPK1 proved suboptimal for mouse RIPK1, notwithstanding high sequence conservation between the two orthologs (Zhang et al., 2025). Notably, in α-synuclein degradation studies, identical E3 ligases (CRBN/VHL/cIAP1) and recruiter ligands (pomalidomide/VH032/bestatin) produced divergent outcomes when paired with different POI ligands (Ser384/Ser308), yielding superior degradation with CRBN and cIAP1, respectively. This suggests potential cooperativity between POI and E3 ligand interactions (Tong et al., 2023; Wen et al., 2023).

The mechanistic underpinnings of these disparities remain incompletely defined but may arise from differential expression of targets or E3 ligases, altered deubiquitinase activity, or other hitherto unidentified regulatory factors. Delineating these determinants is essential to accurately predict cell- and tissue-specific degradation efficacy, and to advance the translational efficacy and safety profile of targeted protein degradation therapeutics.

## Challenges and future directions in PROTAC technology

### Innovations in design and delivery

Despite the contextual uncertainties and challenges discussed, PROTAC technology continues to advance at a remarkable pace, with its research scope expanding from foundational molecular design to encompass delivery systems and novel ligand development. Investigators are not only refining PROTAC delivery modalities, such as enabling efficient peptide-based PROTAC delivery through liposomes and nano-selenium formulations, but are also actively engineering spatiotemporally controllable PROTAC systems (Chan et al., 2024; Zhang et al., 2024a). These include radiation-inducible, hypoxia-activated, and photo-switchable variants that enhance precision and reduce off-target toxicity (Serafini et al., 2025; Wang et al., 2023; Yang et al., 2023).

In parallel, the exploration of E3 ligase ligands has extended beyond classical recruiters. Innovative strategies now leverage protein domains such as HSP90 or N-degrons to engage multiple E3 complexes simultaneously, facilitating synergistic degradation (Dong et al., 2024; ­Gunasekaran et al., 2024). Moreover, PROTAC designs incorporating DNA or RNA aptamers as POI ligands significantly enhance molecular specificity and versatility. Notably, certain ligands traditionally deemed “non-druggable” in conventional inhibitor paradigms—such as short peptide motifs—can exert critical functions within PROTAC architectures, enabling selective target degradation and opening novel therapeutic avenues.

### PROTACs as chemical knockdown tools

Beyond its therapeutic potential, PROTAC technology is emerging as a transformative research tool for biological discovery. By eliciting targeted protein degradation, PROTACs represent a powerful form of “chemical knockdown.” Conventional gene-editing technologies such as CRISPR can introduce off-target mutations, genomic instability, and even cell or organism lethality when targeting essential genes, thereby constraining phenotypic analyses. Compared to genetic methods, PROTACs offer distinct advantages, including reversible action, rapid onset of effect, and the avoidance of permanent genomic alterations. Pioneering work by Sun et al. (2019) demonstrated that systemic administration of the PROTAC molecule RC32 induces robust and reversible degradation of FKBP12 in multiple large animal models, including mice, rats, Bama pigs, and macaques (Sun et al., 2019). Upon compound withdrawal, FKBP12 levels recovered, underscoring the controllability and reversibility of this approach. This modality thus provides a potent new tool for functional genomics and mechanistic studies.

### Navigating the translational pathway: persistent challenges

Despite considerable promise, the clinical and translational development of PROTAC technology confronts several unresolved challenges.

#### Expanding the E3 ligand toolbox

Expanding the E3 ligase ligand toolbox is particularly urgent. Among the over 600 E3 ligases identified in humans, only a handful have been developed for therapeutic use, with clinical research predominantly focused on CRBN and VHL ligands. However, these IMiDs not only face issues of clinical resistance but may also independently induce the degradation of unintended proteins, including their natural substrates IKZF1, IKZF3, and GSTP1 (Petzold et al., 2016). Moreover, the teratogenicity of thalidomide has been linked to CRBN-mediated degradation of developmentally critical substrates such as p63, SALL4, and PLZF (Collins and Scott, 2025). PROTAC design must carefully consider potential off-target degradation and inherent ligand toxicities.

#### Unresolved mechanistic and underdeveloped evaluation

The mechanism of PROTACs remains uncertain. Certain PROTAC molecules fail to effectively degrade POIs, and the specific mechanism behind this remains unclear. Some studies even indicate that high concentrations of PROTAC may cause its POI ligand (small-molecule inhibitors) to lose its inhibitory effect on the POI, instead leading to increased POI expression (Song et al., 2025). These observations underscore the need to deepen our understanding of the UPS and to optimize the rational design of PROTAC molecules.

Furthermore, the evaluation system for PROTACs remains underdeveloped. As a novel therapeutic paradigm, PROTACs require comprehensive and multidimensional assessment criteria that extend beyond conventional metrics (e.g., binding affinity, membrane permeability, and selectivity). Specifically, the efficiency of ternary complex formation, a crucial parameter that directly dictates degradation outcomes, is seldom systematically evaluated. PROTAC-specific properties, such as the hook effect and impacts on global protein homeostasis, must be incorporated into screening workflows. Haid et al. (2023) proposed advanced degradation metrics and computational models integrating parameters such as degradation efficiency and temporal dynamics to better quantify PROTAC efficacy (Haid and Reichel, 2023). Their framework underscores the need to bridge the gap between cellular degradation data and underlying ternary complex thermodynamics. They further emphasized the inclusion of more physiologically relevant models, including primary cells and cross-species comparisons, to improve predictive validity. It is also essential to evaluate downstream pharmacodynamic consequences in addition to degradation efficiency. Reports indicate that certain PROTACs exhibit dual degradation and inhibition functions at low concentrations, or even bind without inducing degradation, resulting in the absence of expected phenotypes (Zhang et al., 2023). Thus, rigorous functional validation is imperative.

#### Targeting beyond the proteome

Looking forward, PROTAC technology may ultimately transcend the degradation of proteins and target non-protein biomolecules. Ubiquitination plays vital roles in diverse biological processes beyond proteostasis, including DNA repair, signal transduction, and immune regulation, and can target molecules such as nucleic acids and lipids. For instance, the CRL4 complex has been reported to ubiquitinate DNA (Scrima et al., 2008), while DTX3L, a member of the DELTEX family, can directly ubiquitinate single-stranded DNA and RNA (Zhu et al., 2024). More recently, ZNFX1 was identified as an atypical E3 ligase that catalyzes ubiquitination of single-stranded RNA during innate immune responses (Grabarczyk et al., 2025). In 2024, Xun et al. developed a miRNA-based PROTAC targeting Lin28 for breast cancer therapy, preliminarily demonstrating the feasibility of RNA-targeting (Xu et al., 2024). Additionally, the ubiquitination of bacterial lipopolysaccharide (LPS) by RNF213 plays a crucial role in anti-infective immunity (Otten et al., 2021). These advances suggest that future PROTAC systems could be designed to ubiquitinate non-protein substrates (e.g., nucleic acids, glycans, or lipids), diverting them toward non-proteasomal fates and unlocking entirely new biological and therapeutic dimensions.

## Conclusion

In summary, PROTAC technology has transitioned decisively from conceptual validation to a promising tool in both clinical development and basic research. As systematically elucidated in this review, the efficacy of a PROTAC is governed not merely by the intrinsic properties of its constituent elements but by the complex interplay among the target protein, the recruited E3 ligase, and the specific biological context. The principle that “context dictates strategy” is of paramount importance. No single E3 ligase represents a universally optimal solution; the strategic selection—whether employing canonical CRBN/VHL ligases for their well-characterized profiles or leveraging emerging ligands such as cIAP or DCAF1 to overcome resistance and expand the degradable proteome—must be ­precisely tailored to the distinct target, tissue, and disease milieu.

As various technical and biological challenges are progressively resolved, the full potential of PROTAC technology will be further unlocked. Continued expansion of the E3 ligase and ligand toolbox is essential to overcome current limitations and enable targeting of novel target classes. Furthermore, the development of controllably engineered systems and comprehensive multi-parametric evaluation frameworks will be crucial for enhancing precision and translational success. Beyond its therapeutic applications, the use of PROTACs as chemical knockdown tools in functional genomics is expected to redefine approaches to biological investigation. Finally, the scope of PROTAC technology is expanding beyond proteins into the new territory of degrading non-protein biomolecules (Fig. 3). By advancing our understanding of context-specific degradation and pursuing these strategic directions, PROTAC technology will continue to serve as a transformative force in both deciphering complex biology and developing new therapeutic strategies.

## Supplementary Material

pwaf107_Supplementary_Data

## Data Availability

The data are all available in the article.

## Abbreviations

AML, acute myeloid leukemia; AR, androgen receptor; CRBN, cereblon; CRL, Cullin-RING ligase; CRL4, Cullin 4 RING ligase; DCAF, DDB1- and CUL4-associated factor; Hyp, hydroxyproline; IMiDs, immunomodulatory imide drugs; IPP, Ile-Pro-Pro; LPS lipopolysaccharide; MDS, myelodysplastic syndrome; MetAP2, methionine aminopeptidase 2; MGDs, molecular glue degraders; MM, multiple myeloma; OVA, ovalbumin; POI, protein of interest; PROTACs, proteolysis targeting chimeras; SAR, structure-activity relationship; UPS, ubiquitin–proteasome system; vHL,von Hippel-Lindau.
