# Supplementary material for: Context matters: E3 ligase–ligand pairing strategies for optimized PROTAC performance
Source: Protein Cell. 2025 Dec 10;17(5):384–98. doi: 10.1093/procel/pwaf107 (PMC13161478; doi:10.1093/procel/pwaf107)
Supplement: pwaf107_Supplementary_Data [file pwaf107_supplementary_data.pdf]

Supplementary Table S1. Summary of Mechanistic Evidence for Ternary Complex Formation.

This table compiles key experimental and computational data from the literature that directly investigate the formation and stability of POI-PROTAC-E3 ligase ternary complexes for the PROTACs discussed in this review.

POI	E3 Ligand	PROTAC Name/ID	Assay/Method Type	Reported Measure	Key Interaction Findings	Reference
H-PGDS	Phenyl-glutarimide	PROTAC-1	Molecular Docking*	N/A	Phenyl-glutarimide moiety interacts with CRBN (His380, Trp382), similar to pomalidomide.	(Osawa et al. 2023)
		PROTAC-2		N/A	Phenyl-glutarimide moiety interacts with CRBN (His380, Trp382), similar to pomalidomide.	
		PROTAC-6		N/A	Fluorine introduction in pomalidomide shows no change in binding mode compared to PROTAC-2. Estimated similar activity to PROTAC-1/2.	
	6-Fluoro-pomalidomide	PROTAC-5		N/A	Phenyl-glutarimide moiety interacts with CRBN (His380, Trp382), similar to pomalidomide. Estimated similar activity to PROTAC-1/2.	
PD-L1	Pomalidomide	9i	Homogeneous time-resolved fluorescence***	IC_{50} = 197.4 nM	Moderate inhibitory activity; functions as a bifunctional degrader-inhibitor.	(Zhang et al. 2024)
		9j		IC_{50} = 219.5 nM	Moderate inhibitory activity; functions as a bifunctional degrader-inhibitor.	
		9i	Microscale thermophoresis***	Kd = 301 nM	Strong binding to PD-L1 despite large molecular size; slightly weaker than POI ligand A56 (Kd = 20.2 nM).	
		9j		Kd = 251 nM	Strong binding to PD-L1 despite large molecular size; slightly weaker than POI ligand A56 (Kd = 20.2 nM).	
		9i	Molecular Docking*	N/A	PEG linker enables stable ternary complex; retains key hydrophobic/halogen bonds with PD-L1; loses some interactions compared to parent ligand A56.	
		9j		N/A	Predicted to form stable ternary complex; maintains key binding interactions with PD-L1; specific interaction profile analogous to 9i.	
RET	Lenalidomide	RD-23	PROTAC-	N/A	Fits into grooves between RET and CRBN;	(Hualong et

			Model*		lenalidomide moiety engages CRBN, LOXO-292 moiety forms H-bonds in RET pocket; Lys716 of RET is a potential ubiquitination site.	al. 2025)
			Molecular dynamics (100 ns) *	Interaction Energy: -96.45 kcal/mol	Forms a more stable ternary complex than RD-4 (lower RMSD, more favorable interaction energy), rationalizing its effective degradation efficacy.	
	Thalidomide	RD-4	Molecular dynamics (100 ns) *	Interaction Energy: -87.76 kcal/mol	Forms a less stable ternary complex than RD-23 (higher RMSD, less favorable interaction energy), consistent with its lower degradation efficacy.	
ASK1	VHL ligand	dASK1	Induced Fit Docking (Schrödinger) *	N/A	Thalidomide moiety forms H-bonds with CRBN (His378, Trp380, Asn351, Ser379, Trp386); Selonsertib moiety forms H-bonds with ASK1 (Lys709, Gln756, Asp822).	(Sarkar et al.)
			MM/GBSA*	Favorable Binding Free Energy	Intermediate linker length (~12 Å distance) yields most stable ternary complex; linker's ethereal oxygen interacts with ASK1 Arg705; optimal linker length is critical for degradation efficiency.	
MDM2	VHL ligand	V10	Molecular Dynamics (100 ns) *	RMSD ~2.0 Å (avg)	Ternary complex MDM2/V10/VHL remains stable throughout simulation (max RMSD < 3.6 Å). Complex was constructed by linking pre-docked MDM2 and VHL binary structures.	(Li et al. 2024)
HSP90	Lenalidomide	lw13	Molecular Docking*	N/A	Active warhead binds Hsp90 ATP site (key H-bonds with ASP77, ASN35, TYR123); lenalidomide moiety binds CRBN differently from parent; linker ester group interacts with CRBN SER42.	(Liang et al. 2024)
			Molecular Dynamics (6 μs) *	RMSD ~0.6 nm (after 2.5 μs)	Ternary complex Hsp90/lw13/CRBN is highly stable, confirming simultaneous binding to both Hsp90 and CRBN.	
PI3Kδ	VHL Ligand	B14	Molecular Docking*	N/A	PI3Kδ ligand forms covalent bond with Lys779 and key H-bonds (Val828, Asn836, Asp911); VHL ligand forms H-bond with His115 and vdW contacts (Trp88, Tyr98, Trp117); flexible decanoyl linker is suitable.	(Yuan et al. 2025)
KSP	Thalidomide	21	PROTAC-Model*	N/A	S-trityl-l-cysteine moiety binds KSP allosteric site (H-bonds with Arg221, Glu116); thalidomide group binds CRBN (H-bonds with Trp380, His378, Asn351),	(Zhao et al. 2025)

					suggesting effective ternary complex formation.	
BRD9	DCAF1 Ligand	DBt-5	Surface Plasmon Resonance***	Required higher conc. for max SPR response than DBt-10.	Maximal ubiquitination initial velocity coincided with max ternary complex formation.	(Schröder et al. 2024)
			NanoBRET (Target Engagement) **	Potent BTK engagement in intact/permeabilized cells.	Superior membrane permeability compared to DBt-10.	
		DBt-10	Surface Plasmon Resonance***	Lower conc. for max SPR response; higher max response than DBt-5.	Formed more stable ternary complex. Maximal ubiquitination initial velocity occurred at higher conc. than complex formation, but absolute rate was higher than DBt-5	
			NanoBRET (Target Engagement) **	Reduced BTK engagement without membrane permeabilization.	Inferior membrane permeability compared to DBt-5.	
BCL-2	Pomalidomide	15	AlphaLISA***	Forms complex with BCL-xL, but not with BCL-2.	Selectively forms BCL-xL/PROTAC/CRBN complex (less than XZ739), and fails to form BCL-2 complex, explaining its degradation selectivity.	(Bricelj et al. 2024)
		19		Forms complex with BCL-xL, but not with BCL-2.	Selectively forms BCL-xL/PROTAC/CRBN complex (less than XZ739), and fails to form BCL-2 complex, explaining its degradation selectivity.	
		XZ739 (Control)		Able to form ternary complex with BCL-xL (strong signal).	Used as a positive control; forms a more robust BCL-xL/PROTAC/CRBN complex compared to PROTACs 15 and 19.	
GPX4	cIAP Ligand	18a	ICM PROTAC Modeling*	N/A	POI Ligand warhead binds GPX4 via Cys66; cIAP1 ligand forms H-bonds with Glu163, Arg308, and Gly306. Confirms simultaneous binding to both proteins.	(Song et al. 2024)
			Molecular Dynamics (200 ns) *	RMSD < 3 Å (100-200 ns)	The ternary complex reaches a stable conformation after initial equilibration, with RMSD maintained below 3 Å, supporting the stability of the predicted structure.	
FGFR2	Thalidomide	28e	Molecular Docking*	N/A	Constructed by docking ligand 20 to FGFR2 and pomalidomide to CRBN, then	(Hu et al. 2024)

			linking. Serves as the initial structure for MD simulation.
	Molecular Dynamics (100 ns) *	RMSD 3-5 Å (after 40 ns)	The ternary complex FGFR2/28e/CRBN reaches a relatively stable conformation after 40 ns, with RMSD fluctuating between 3-5 Å.

*Computational Modeling； **Cellular Indirect Evidence; ***Biophysical/Binding Assay
